# The Clinical Society of Maryland

**Published:** 1901-01

**Authors:** 


### SOCIETY REPORTS.

---

### THE CLINICAL SOCIETY OF MARYLAND.

BALTIMORE, November 16, 1900.

In the absence of the president, the meeting was called to order by Dr. H. B. Jacobs, vice-president and chairman of the Executive Committee.

Dr. Samuel Amberg read a paper on "Simple Goitre in Infancy," and exhibited a number of very interesting cases.

In discussing the question, Dr. Harry T. Marshall said: It is interesting in connection with the loss of the thyroids to consider the number of instances in which the thyroid is enlarged. Dr. Halsted, some years ago, operated upon a number of dogs, taking out part or all of the thyroid and in not a single case did he fail to get a large growth of the accessory thyroids. It is probable that in human beings the same conditions would attend, for when there is an enlargement of the normal thyroid, there is usually some enlargement of the accessory glands. Such enlargements have been found in the trachea and even in the bronchi, causing by obstruction the symptoms of asthma.

*Dr. Jacobs:* An interesting point in the therapeutics of simple goitre is that long before the thyroid extract was discovered it was known that iodine would benefit such conditions, and since it has become known that thyroid extract has a beneficial effect upon them it has been discovered that the thyroid extract contains iodine. It is an example of the occasional importance of empiric methods of treatment.

An eminent authority in Geneva has said that he invariably puts these patients on thyroid extract and feels sure he can always say that the goitre will, at least, be reduced in size.

*Dr. A. L. Hodgdon:* Apropos of Dr. Jacobs's remarks, I would like to say that I have found in these cases of goitre that iodine administered cataphorically will reduce their size.

"Amblyopia and Squinting Eye Benefited by Cataract Operation." Exhibition of Patient. By Dr. Herbert Harlan.

It is well known that in the majority of cases of squint one of the eyes is more or less amblyopic, and it is a question whether it is the amblyopia that causes the squint or whether the amblyopia results from the lack of the use of the eye caused by the squint. I think there is very little doubt that there are cases in support of both sides of this question. There was one very interesting case reported some time ago of a boy, thirteen or fourteen years old, whose vision had been very carefully tested and recorded, and who afterward had to have his good eye enucleated as the result of an injury by a piece of steel. In the course of a week after the operation he began to see out of the eye which had been amblyopic and the vision continued to improve until it became very good. The patient I wish to exhibit to-night has a somewhat similar history.

He is a man of sixty-two years, whose left eye turned sharply in towards the nose, and he thinks it has been so since early infancy. At the time I first saw him he was very markedly jaundiced and had just recently been operated upon for gall-stones. The squinting eye had a mature cataract and the other one an incipient cataract. It occurred to me to operate on the squinting eye while the other cataract was in the process of maturing. I did so, and since that time the cataract in the right eye, which was the straight one, has entirely matured. He assures me positively that ever since he knew anything about it, he has had nothing more thon light perception in the left eye. A simple extraction was performed and he only remained in the hospital nine days. So long as the other eye was good enough to allow him to move about, the vision in the amblyopic eye improved very little, but, after the cataract in the good eye had matured the vision in the eye that had been operated upon improved rapidly. At first he had a great deal of difficulty in turning the eye out, and on that account I performed a double tenotomy. He now has vision of 15/200 in the amblyopic eye, and as there is a slight capsular deposit a capsulotomy may give him better vision still.

This is probably a clear case of sight restored to an amblyopic eye and seems to prove that that eye at least was amblyopic on account of lack of use. (Exhibition of patient.)

DISCUSSION.

*Dr. H. O. Reik:* The case which Dr. Harlan has just reported is not only extremely interesting in itself, but has an important bearing on the theory of amblyopia exanopsia. It has long been a subject for debate, whether in these cases of strabismus the amblyopia constitutes a congenital lesion or is the result of long-continued suppression of the visual image.

Dr. Harlan referred to the case of the file-worker, reported by Dr. Johnson several years ago, in whom the loss of the good eye as result of an accident was followed by rapid and complete restoration of normal vision in the amblyopic eye. Since then Dr. Johnson has reported two other cases presenting strong clinical evidence in support of the view that amblyopia can follow non-use of an eye. One of these was, in effect, similar to the above, I believe; recovery of vision in the bad eye when compelled to use it because of loss of the good eye. The third case was valuable information from another point of view. The patient received an injury to the brow and eye, involving the eye muscles in such a way that binocular fixation because impossible. For some time after the injury there was troublesome double vision and several examinations showed full normal vision in both eyes. The patient learned, however, to suppress the image in the squinting eye, the diplopia disappeared, and a careful examination several years later showed that the vision in this eye had fallen from 30/20 to 20/100.

Javal, Risley and others have published similar cases, and, in face of the clinical evidence now collected, it seems impossible to doubt any longer the existence of an amblyopia exanopsia.

*Dr. R. L. Randolph:* I am very much interested in Dr. Harlan's paper, and I am reminded of a case recently reported by a German, I believe, within the last three months. Klein, I think, also reported a similar case some three years ago. The case recently reported was a boy, eleven years old, who had always squinted strongly with his right eye. The left eye was wounded by a piece of wood, and as a result ulceration of the cornea followed with prolapse of the iris and his vision was reduced to light perception. It was then thought wise to exercise the squinting eye, which only possessed ability to count fingers at five feet. Within two weeks he was able to count fingers at twenty feet, and within twelve months the vision had gone up to 20/30 and he could read the finest print with the aid of a weak concave glass.

These cases seem to confirm Priestly Smith's idea concerning the early treatment of squint. He has laid down rules for systematically exercising the squinting eye while binding up the good one. I never operate upon these cases of squint in children until they can help me by such exercise.

*Dr. Harlan:* Have you ever gotten any benefit from tying up one eye?

*Dr. Randolph:* I cannot say that I have.

*Dr. Hiram Woods:* The question of an improvement in a squinting eye when it is compelled to work is an interesting one. There are cases in which no amount of treatment will develop the sight. One of the most remarkable cases I have ever seen occurred a year ago in a young lawyer in Baltimore. He had a normal right eye with a very small error of refraction. There was a latent squint which was an internal one. He had a high error of refraction in the left eye, his best vision being 20/50, and he discarded the left altogether in the fusion of stereoscopic pictures. I persuaded him to exercise that eye, and in six months his vision improved to normal, and he had stereoscopic vision for the first time in his life.

Now, it is a question in my mind, when these cases develop sight rapidly, whether it is an actual improvement in sight or a becoming conscious, on the part of the patient, of the sight he has not previously noticed. It is a common thing in testing refraction to have the patient stop short at 20/50 or thereabout, and swear he cannot read any further, but by gentle coaxing you bring him to 20/40 or 20/30, or some point far above what he thinks he is capable of. So, in my case, it was a question whether the vision had improved, or whether the patient had been taught to work up to his abilities.

So far as this rapid improvement of vision in Dr. Harlan's case is concerned, I wish we had something more to act on than simply the patient's statement. As long as one has an eye that gives him ability to walk about, and a perfect eye on the other side, the degree of vision existing in the bad eye will not impress him very much; but let something happen to destroy the good eye, and he is compelled to use the bad eye, and he comes to appreciate it very much more, and there follows a development right up to the limit of which that eye is capable. This man's whole mental attitude is very different now from what it was when he had a perfect eye on the other side. This is a phase of the matter that throws some doubt on the rapid development of visual power; the possibility of the eye really having more power of vision than the individual appreciates, has to be considered. To test the eye carefully now and then again a year hence would, to my mind, give much more valuable information than we have in the patient's statement due to the sudden change.

*Dr. Harlan:* I am perfectly satisfied in my own mind in regard to this question ot amblyopia exanopsia. In this case the interesting point was the length of time the squint and amblyopia had existed. I grant the force of Dr. Woods's objections, but my experience has been, both with children and adults, that though they say the eye is not good, they claim to see more than they actually do when I come to test them. There is no doubt this man's vision has improved very much since the operation. After the operation he only counted fingers at close range until the sight of the other eye faded out, and then the amblyopic eye began to show a rapid improvement.

"An Ambulatory of Typhoid Fever." Exhibition of specimens. Dr. Harry T. Marshall.

The interesting part of this case is its clinical history. The patient was admitted to the hospital November 9th, in an irrational condition, and his history was unsatisfactory on account of his mental confusion. He said he had been suffering for three weeks with headache, loss of appetite and dizziness. During these three weeks he had been wandering about the streets, more or less, continuously, and sleeping out of doors at night. His family found him three or four days previous to his admission into the hospital, but he refused to go to bed. Examination showed a sallow skin, pale mucous membranes and very poor pulse. The spleen was palpable and abdomen tympanitic. While in the hospital he was given subcutaneous injections of salt solution, stimulants, and turpentine stupes for the tympanites. He did not respond to treatment, but had a hemorrhage from the bowels, which was repeated at intervals of a few hours until he had five, shortly after which he died. His temperature was 101.4 on admission, and 104 at the time of death. The pulse varied from 104 to 124, and respiration from 24 to 32.

The condition at autopsy was typhoid at the end of the second or beginning of the third week. The interesting feature of the case is, that it was a case of ambulatory typhoid, walking about the streets in spite of the most intense typhoid lesions in the upper bowel. He had no diarrhea, so far as we could ascertain, but he could readily have spread the germs very widely. Another very interesting fact is that he had five hemorrhages within twenty-four hours without any change in his temperature, which remained persistently high.

### DISCUSSION.

*Dr. John S. Fulton:* I would like to say a word as to the great importance of these cases, having had under observation an outbreak that illustrates the great damage this kind of a case can do. On the 5th of September a case of typhoid fever died at a farm house. The family had been assisted in the care of this patient by the wife of a dairyman on the adjacent farm. A short while afterward this woman and her son began to complain. The physician attending them diagnosed the trouble as "summer grip," and still maintained that diagnosis. I saw the boy and woman both recently, and both have the appearance of convalescents from typhoid fever. The boy milked the cows, some 15 or 20, daily, and the mother attended to the cans. On the 8th day of October this boy went to bed for the first time and remained there but a few days; the mother continued her duties.

On the 11th of October a case of typhoid occurred on the route of this milkman, in a town of 2,500 inhabitants, and between the

11th and 27th of the month there occurred in that town 35 cases, and of those 34 took milk from this man. Their drinking water was either the town supply, or, in about a dozen cases, private wells. The largest daily incidents of typhoid fever cases occurred on the 18th, six being reported for that day. The milkman was compelled to stop serving milk, and since the 27th of October the number of typhoid cases on his route has reached about 43, making in all 78. Of course a few more are due. The one case that was counted as not having gotten the infection from that milk has since been proven to have gotten the infection from the same farm, although she did not drink milk.

I mention this because it seems to illustrate so well a malignant infection from a case of typhoid that had not been compelled to stay in bed at all. An older boy in this same family is now in bed with a severe attack of the same disease, which is likely to prove fatal. If it is "summer grip," it is a bad series of cases.

*Dr. J. M. Craighill:* I think it is sometimes quite hard to recognize these cases of ambulatory typhoid. Any physician who is doing dispensary work sees cases almost daily in which it is hard to make a positive diagnosis. Only to-day at the University Hospital dispensary we saw a man that we think has typhoid fever but we could not be sure. An examination of the blood gave no positive result. He had an enlarged spleen, was tender about the iliac region on the right side, and we thought we distinguished some rose spots on the abdomen. In a day or so, probably, we can make a diagnosis, although it will still be early to decide from the Widal reaction.

Rather an interesting set of cases came under my care in the hospital recently. Three sailors were admitted from the same ship. Two of them had typical malarial symptoms and showed the plasmodium in the blood. The third had typhoid symptoms and gave the Widal reaction, but after three or four days the temperature took on the malarial curve, and, responding quickly to quinine, he went out with the rest of the patients in a short time.

*Dr. H. B. Jacobs:* It seems to me this is a most interesting case. It is interesting to know that a man could walk about with such a lesion as he had in his ileocecal valve. Then the ability of that man to spread the disease is certainly very great. When one thinks of the more recent results of the careful examination of the urine, which show that it, too, is full of the typhoid germs, and considers that it is likely to be voided in any place where such a man happens to be, it is manifest that the danger of spreading infection is greatly increased.

*Dr. Randolph:* I would like to ask Dr. Marshall what would have been the objection to operating after the first hemorrhage in this case, and what are the symptoms that determine one to operate in a case of this character.

*Dr. Marshall:* I did not see this case in life, but suppose it was not operated upon because of its peculiar history. There was little or no distention of the abdomen, the leucocytes were down to 3500 and did not rise at all, and there were no localizing symptoms in the abdomen at all. There was nothing here that would lead one to suspect perforation.

In regard to the most important signs for determining to operate, perhaps some of the surgeons present can tell you better than I can. The general condition of the patient would, of course, count for much in considering the question of operating.

H. O. REIK, M.D., Secretary,
5 N. Preston St.

---

### "SWALLOWING" A DIAMOND.

We are indebted to Surgeon-General Bidie, C.I.E. (I.M.S. retired), for sending us a local report of a curious case recently tried in Calcutta. A young man, having provided himself with a new suit of clothes, walked into a jeweller's shop and asked to look at a parcel of diamonds. He went to the window for better light, and presently it was discovered that the largest diamond, valued at Rs. 10,000, had disappeared. The gem did not appear, and it was suspected that it lay concealed in the man's throat. A skiagram was taken and revealed a foreign body in the throat which could not be dislodged. At the trial an old criminal swore that the concealment of stolen articles in the throat was a well-known habit of Indian thieves. An artificial dilatation of the pharynx is produced by the means of a round leaden bullet, which is "swallowed" and brought up several times a day until a pouch is made which fulfils for stolen articles—coins and jewels—the same function as the crop in birds and rumen in ruminants.—*British Medical Journal.*

